# Evolving thresholds for liver transplantation in hepatocellular carcinoma: A Western experience

**DOI:** 10.1002/ags3.12316

**Published:** 2020-02-11

**Authors:** Michelle R. Ju, Adam C. Yopp

**Affiliations:** ^1^ Division of Surgical Oncology Department of Surgery University of Texas Southwestern Medical Center Dallas Texas

**Keywords:** hepatocellular carcinoma, liver transplant selection, organ stewardship, transplant criteria, transplantation

## Abstract

Hepatocellular carcinoma (HCC) is the second leading cause of cancer‐related deaths worldwide. Once considered an experimental treatment with dismal survival rates, liver transplantation for HCC entered a new era with the establishment of the Milan criteria over 20 years ago. In the modern post‐Milan‐criteria era, 5‐year survival outcomes are now upwards of 70% in select patients. Liver transplantation (LT) is now considered the optimal treatment for patients with moderate to severe cirrhosis and HCC, and the rates of transplantation in the United States are continuing to rise. Several expanded selection criteria have been proposed for determining which patients with HCC should be candidates for undergoing LT with similar overall and recurrence‐free survival rates to patients within the Milan criteria. There is also a growing experience with downstaging of patients who fall outside conventional LT criteria at the time of HCC diagnosis with the goal of tumor shrinkage via locoregional therapies to become a candidate for transplantation. The aim of this review article is to characterize the various patient selection criteria for LT, discuss balancing organ stewardship with outcome measures in HCC patients, present evidence on the role of downstaging for large tumors, and explore future directions of LT for HCC.

## Graphical abstract

Hepatocellular carcinoma (HCC) is the second leading cause of cancer‐related deaths worldwide. Once considered experimental treatment with dismal survival rates, liver transplantation for HCC entered a new era with the establishment of the Milan criteria over 20 years ago. The aim of this review article is to characterize the various patient selection criteria for liver transplantation, discuss balancing organ stewardship with outcome measures in HCC patients, present evidence on the role of downstaging for large tumors, and explore future directions of liver transplantation for HCC.

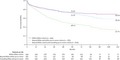

## 1 INTRODUCTION

Hepatocellular carcinoma (HCC) is the sixth most common cancer and the second leading cause of cancer‐related deaths worldwide.^1^, ^2^ HCC most commonly occurs in a background of chronic liver disease with or without cirrhosis.^3^, ^4^, ^5^ Prognosis is not only dependent on tumor burden, but on underlying liver function and patient performance status.^6^ Surgical resection is the standard of care for patients without underlying liver disease and with preserved liver function who develop HCC. In select patients, 5‐year survival can approach 70% for those without cirrhosis and even 60% for those with Child‐Pugh A cirrhosis. However, due to heterogeneity of the patient population and low utilization of HCC screening, only 10%‐37% of patients are candidates for surgical resection at initial HCC diagnosis.^7^, ^8^, ^9^

Liver transplantation (LT) for HCC has developed dramatically over the past few decades with new criteria and prognostic factors that are continuing to evolve. In 2017, the number of liver transplants performed in the United States was at an all‐time high, with 22% of all liver transplants done for underlying HCC, making HCC the most common indication for LT.^10^ Liver transplantation is an especially attractive option for the treatment of HCC in cirrhotic patients as it simultaneously addresses the cancerous lesion and the underlying liver dysfunction, which is at risk for developing new HCC lesions.^11^ In patients who have undergone surgical resection for HCC, the 5‐year recurrence rate is significantly high, with >50% of patients exhibiting locoregional recurrence.^12^, ^13^ The most significant risk factors for recurrence after HCC resection are the presence of underlying cirrhosis and active hepatitis.^14^ Patients with cirrhosis are thought to frequently have genetic alterations that represent a field defect putting the entire liver parenchyma at risk for development of cancer.^15^ Compared with surgical resection, the recurrence rate of HCC after LT is estimated to be around 15%‐20%.^16^, ^17^, ^18^

Thus, given that LT both removes detectable/undetectable tumors and preneoplastic lesions that are present in the cirrhotic liver, as well as addressing the underlying cirrhotic liver parenchyma, LT is now considered the optimal HCC treatment for patients with advanced (Child‐Pugh B or C) cirrhosis. Better preoperative assessment of liver function, improved accuracy of cross‐sectional imaging studies, and surgical technical progress are key factors that have led to reduced mortality with LT, and 90‐day mortality is currently estimated at around 5%.^19^ In selected patients, liver transplantation can offer an expected 5‐year survival around 70%.^20^

Although the benefits of LT for patients with HCC are clear, there is ongoing debate surrounding the selection of patients who would be best served by LT and how patients who initially fall outside of recognized criteria for transplant at the time of diagnosis should be managed. The aim of this review is to characterize the various patient selection criteria for LT, discuss balancing organ stewardship with outcome measures in HCC patients, present evidence on the role of downstaging for large tumors, and explore future directions of LT for HCC.

## 2 HISTORICAL PERSPECTIVES

The first liver transplant performed for HCC was completed in 1967 by Thomas Starzl. Due to a lack of guidelines regarding organ allocation in a nascent surgical field, decision making regarding which patients with HCC received liver transplantation was at the discretion of the treatment team. Unfortunately, the lack of recognized organ allocation criteria for HCC patients led to poor outcomes and a temporary moratorium in the United States for liver transplant with HCC acts as an indication of these outcomes. Prior to the global acceptance of organ allocation criteria, the two largest series describing liver transplantation for HCC were published by Bismuth et al^21^ and Iwatsuki et al.^22^

In a report of the European Liver Transplant Registry, Bismuth detailed the results of 32 European centers performing a total of 1218 liver transplants, with over two‐thirds of those cases done after 1984.^21^ HCC accounted for 18% of LT indications in this report, with the majority of patients with HCC selected for LT because they were not candidates for resection secondary to significant tumor burden or underlying liver dysfunction. The 30‐day mortality rate was 30% for all liver transplant patients and 24% for HCC liver transplant patients specifically, which was the lowest rate of any group. The overall 2‐year survival for HCC patients undergoing liver transplantation was 30%.

Iwatsuki et al^22^ published an early description of the American HCC liver transplantation experience in 1985. Five‐year survival rates were well under 30%. Seventy‐two per cent of patients recurred, with 69% of those recurrences occurring <1 year after transplantation. The grafted liver and the lung were most commonly affected by tumor recurrence. Several cases of patients receiving LT, who shortly thereafter were found to have metastatic disease, were described. The dismal long‐term survival rates in these early series were largely related to the lack of any a priori criteria for determining which patients with HCC should receive liver transplantation.

## 3 THE DEVELOPMENT OF MODERN‐DAY CRITERIA

The development of the milan criteria (MC), introduced by Mazzaferro et al^23^, ushered in a new era of liver transplantation for HCC. The Milan criteria demonstrated that liver transplantation in patients with HCC with the following criteria had significantly improved outcomes compared to patients transplanted for HCC outside of the criteria: (a) single tumors with a diameter <5 cm; (b) no more than three tumors, each ≤3 cm in size; (c) no vascular invasion; and (d) no extrahepatic involvement. This series included 48 patients undergoing LT between 1991‐1994 with most patients (94%) having underlying cirrhosis. The 4‐year overall survival rate was 75% and recurrence‐free survival rate was 83%, a dramatic improvement from pre‐1996 series. This study highlighted that appropriate preoperative selection of patients was critical to improving survival after transplantation. In the 35 patients (73%) whose pathological examinations confirmed that they had tumors which met the pre‐determined Milan criteria, 4‐year overall survival was 85% with recurrence‐free survival of 92%. However, in the 13 patients (27%) who were assigned an inaccurate stage prior to LT and had tumors that exceeded those limits, 4‐year overall survival was only 50% and recurrence‐free survival was 59%.

The Milan criteria were quickly adopted worldwide, as well as built into a prioritization tool in the United Network of Organ Sharing (UNOS). However, as experience with LT for HCC continued to grow, critics voiced concerns that the Milan criteria may be too restrictive, excluding some patients from undergoing LT who would benefit from transplantation. Critics challenged these criteria, saying they were too strict because they excluded specific subgroups with meaningful, albeit slightly lower, opportunities to benefit from LT, as demonstrated by mostly single‐institution studies.^24^, ^25^, ^26^, ^27^, ^28^ It was also pointed out that cross‐sectional imaging techniques had improved to enable detection of very small (<1 cm) lesions which were undetectable when Milan Criteria were first written.

**Figure 1:**
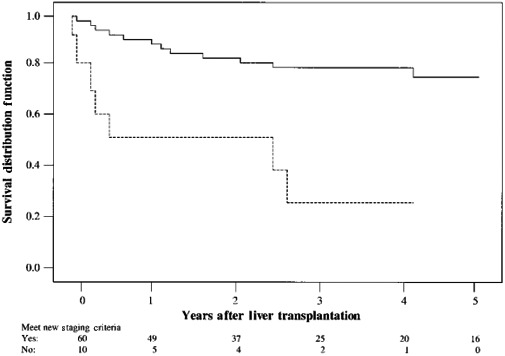
Survival probabilities according to the UCSF criteria based on pathology of liver explants. Meeting criteria: (solid line), yes; (dashed line), no. Reprinted from Yao et al^29^ with permission from John Wiley and Sons

In 2001, Yao et al^29^ published what is now known as the UCSF criteria. Their group identified a small subset of patients with HCC who had tumors exceeding the Milan criteria but still had comparable survival. They defined a new set of criteria as solitary tumor ≤6.5 cm or ≤3 nodules with the largest lesion ≤4.5 cm and total diameter ≤8 cm. Patients meeting these expanded criteria based on pathologic examinations of the explanted liver (n = 60) had a 5‐year overall survival rate of 75% in this series. When these same criteria were applied to the preoperative staging of HCC based on radiologic findings in 45 patients, 37 patients who met the criteria had a 5‐year overall survival rate of 84% (Figure 1). Preoperative HCC stage was accurately estimated in 76% of cases and underestimated in 16%.

Yao et al^30^ subsequently published a validation of the UCSF criteria in 2007 by applying these criteria for LT based on pretransplant imaging in 168 patients. The 5‐year recurrence‐free survival rate was 81%. Tumor under‐staging was observed in 20% of patients, and the 5‐year recurrence‐free probability for this group was 60% compared to 97% of those who were accurately staged as falling within the criteria. They estimated that the UCSF criteria offered the potential benefit of LT to an additional 5%‐20% of patients with HCC who would have otherwise been excluded under the more restrictive Milan criteria, with comparable overall survival and recurrence‐free survival rates.

**Figure 2:**
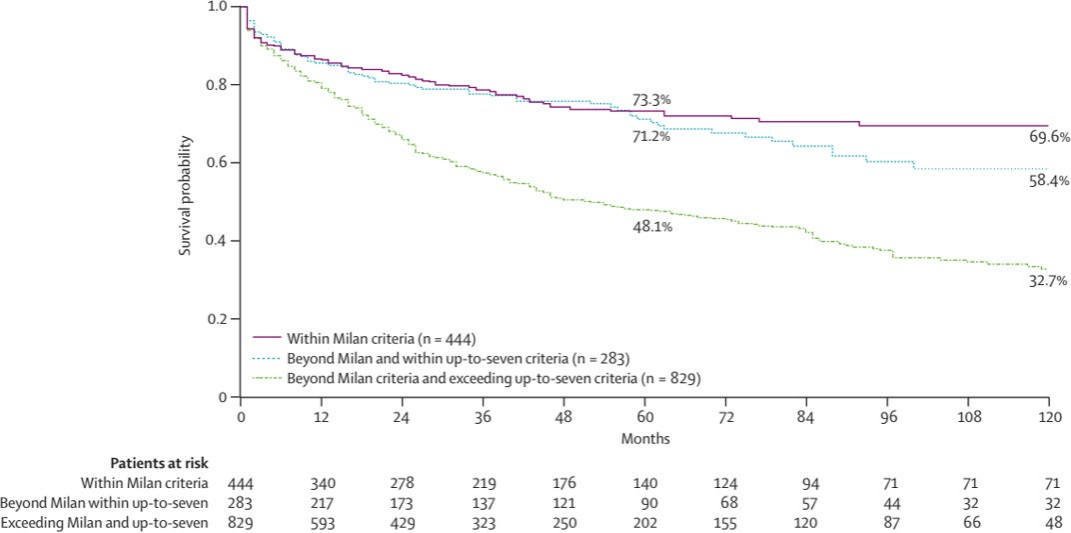
Survival for patients within Milan, beyond Milan but within up‐to‐seven, and exceeding both sets of criteria. Reprinted from Mazzaferro et al,^31^ Copyright 2009, with permission from Elsevier

With studies now showing that patients with larger tumors and larger total tumor size were achieving 5‐year survivals similar to patients meeting the Milan criteria, there was an increased interest in exploring the survival of patients with tumors exceeding previously defined limits. Mazzaferro et al^31^ published a study in 2009 based on a collective database among 36, mainly European, centers which identified a subgroup of patients with HCC exceeding Milan criteria who achieved a 5‐year overall survival of at least 70% (i.e. similar to previously demonstrated overall survival rates for patients meeting Milan criteria). A total of 283 patients fell within what they defined as the up‐to‐seven criteria: HCCs with seven as the sum of the size of the largest tumor (in centimeters) and the total number of tumors, with no microvascular invasion. This group achieved a 5‐year overall survival of 71.2% after LT. Patients exceeding the up‐to‐seven criteria, plus patients with microvascular invasion who were beyond MC and within the up‐to‐seven criteria, had a 48% 5‐year overall survival rate (Figure 2).

In a departure from “math‐based” to “biology‐based” selection criteria, the extended Toronto criteria were introduced by Dubay et al^32^. The extended Toronto criteria offer LT to patients with HCC confined to the liver (no restrictions on tumor size or number), high‐performance status, no vascular invasion on imaging, and biopsy confirmation that the dominant lesion lacked a poorly differentiated histology. Patients exceeding Milan criteria had 5‐year overall survival of 72% vs 70% for patients meeting Milan criteria and 66% vs 70% disease‐free survival, respectively (not statistically significant). They found significant discordance between pre‐LT imaging and liver explant pathology, with 30% of patients in the Milan criteria group being under‐staged and 23% of patients in the expanded group being over‐staged.

**Figure 3:**
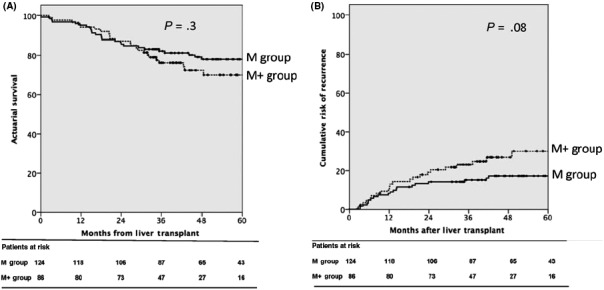
A, Overall survival of validation cohort from Sapisochin et al 2016; B, cumulative risk of recurrence of validation cohort. Reprinted from Sapisochin et al,^33^ with permission from John Wiley and Sons

Sapisochin et al^33^ later published a validation study of the extended Toronto criteria in 2016 in a prospective cohort of 243 patients (57% within MC, 43% beyond MC, 28% of whom met UCSF, and 72% beyond UCSF). The median time to LT was 6.4 months, with a 14% drop out rate, predominantly for tumor progression. The overall survival for the group exceeding MC was similar to that of the MC group at 1‐, 3‐, and 5‐years: 94%, 76%, and 69% vs 95%, 82%, and 78% (*P* = .3; Figure 3). The incidence of recurrence was higher in the group exceeding MC (25.6% vs 16.1% for the MC group, *P* = .09) with median time to recurrence of 12.8 months. Despite the pretransplant biopsy, 8% of patients exceeding MC had a poorly differentiated tumor at explant pathology. The inclusion of mandatory biopsy in the selection criteria raises some concern due to large variations in the accuracy of tumor grading ranging from 27.5%‐84.8% depending on lesion size and heterogeneity^34^ and the possibility of needle‐tract seeding (mean incidence 3%, up to 8%)^35^ (Table 1).

**Table 1:** Tumor criteria and overall survival statistics after LT for HCC

Criteria	1‐year survival	3‐year survival	4‐year survival	5‐year survival
Milan	—	—	75%	—
UCSF	90%	—	—	75.2%
Up‐to‐seven	—	77.7%	—	71.2%
Extended Toronto	94%	76%	—	69%

As interest grows in the development of biology‐based criteria, multiple new models have been introduced in recent years. The Metroticket 2.0 model, created by Mazzaferro et al,^36^ utilizes AFP level, tumor size, and tumor number to determine the risk of HCC‐specific death after LT. The Metroticket model outperformed Milan, UCSF, and up‐to‐seven criteria in predicting 5‐year survival after transplantation. The use of tumor‐marker des‐gamma‐carboxy prothrombin (DCP) as a predictor of risk of HCC recurrence after LT has also recently gained interest. In a meta‐analysis, DCP was a useful predictive factor indicating a 5‐fold increased risk for recurrence after transplantation.^37^ These markers, such as AFP and DCP, may represent a way to further refine selection criteria for LT based on tumor biology.

## 4 CURRENT UNOS ALLOCATION POLICIES, WAITLIST TIMES, AND ORGAN STEWARDSHIP

The Organ Procurement and Transplantation Network (OPTN)/Scientific Registry of Transplant Recipients (SRTR) 2017 Annual Data Report found a continued growth in the number of new waitlist registrants (11 514 in 2017 vs 11 340 in 2016 and 10 636 in 2015) and a continued increase in the transplant rate (51.5 per 100 waitlist‐years).^10^ However, although decreasing with time, a significant gap continues to exist in transplant receipt rates for HCC vs non‐HCC candidates. The gap was larger among women, with a transplant rate 2.1‐fold higher for HCC vs non‐HCC candidates (97.0 vs 45.4 per 100 waitlist‐years). Among men, the transplant rate was 72% higher for HCC vs non‐HCC candidates (89.4 vs 52.1 per 100 waitlist‐years).

In the USA, starting in 2002, patients with HCC receive priority listing for LT if they have UNOS T2 lesions (single tumor between 2‐5 cm or 2‐3 tumors each ≤3 cm without vascular invasion or extrahepatic spread). This system was developed to give patients with HCC an equal opportunity for transplantation via a “priority Model for End‐Stage Liver Disease (MELD) score” beyond their degree of hepatic decompensation because MELD cannot accurately predict mortality in HCC.^38^ The initial prioritization granted HCC patients 29 MELD points for T2 tumors and 24 points for T1 HCC tumors (one lesion ≤1.9 cm). However, some argued that this prioritization had gone too far and was no longer equitable to non‐HCC patients awaiting LT.^39^ This has led to multiple reiterations of MELD prioritization for HCC with the current allocation of “natural” MELD points for the first 6 months following listing for transplantation and then an allocation of 28 points with a cap of 34 points.^40^

Given the extremely limited availability of organs for transplantation in the USA, expansion of selection criteria for LT in HCC must be weighed carefully with regards to the limited supply of organs and the long‐term outcomes of patients undergoing LT for HCC outside of established allocation criteria. In an attempt to determine the optimal benefit of LT for HCC where HCC patients undergoing transplant would not harm patients listed for liver transplant for non‐HCC etiologies, Volk et al^41^ created a Markov model comparing survival benefit of LT for a patient with HCC exceeding MC vs the harm caused to other patients awaiting transplant. They concluded that a 61% 5‐year overall survival rate was needed to outweigh harms to non‐HCC patients awaiting LT. Their conclusions, although a bit dated with ever‐changing organ allocation policies, serve as a guideline regarding the stewardship of liver allocation.

**Figure 4:**
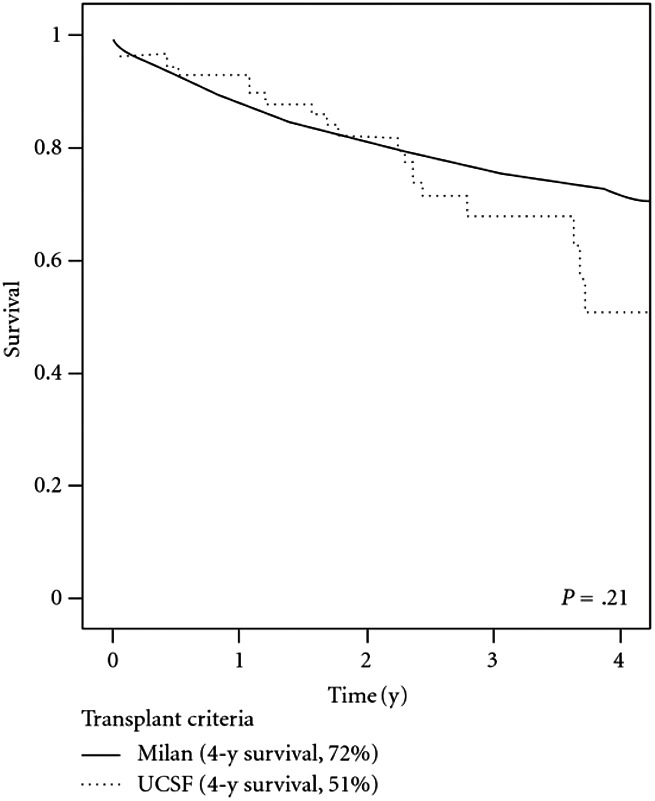
Survival analysis of HCC patients treated with LT stratified by Milan and UCSF criteria. Reproduced without changes from Patel et al^40^ under the Creative Commons Attribution License

Patel et al^42^ conducted a retrospective review of the UNOS register in 2012 examining outcome differences between patients within Milan criteria and those exceeding Milan criteria but meeting UCSF criteria. A total of 1972 patients were included, with the vast majority meeting MC based on imaging studies at the time of LT (97%). Only 59 patients (3%) were outside MC but within UCSF criteria. The 1‐, 3‐, and 4‐year survival rates for the Milan cohort was 89%, 76%, and 72% vs 91%, 68%, and 51% for the UCSF cohort (Figure 4). Although survival was numerically lower for patients outside Milan criteria but within UCSF criteria vs patients within MC, the difference was not statistically significant (*P* = .21).

While there is conflicting data for expanded criteria outside of UCSF criteria, overall these patients appear to have higher recurrence and lower overall survival. The potential benefit from LT to patients exceeding expanded criteria may be outweighed by potential harms to others on the waitlist. Further higher‐quality, large‐scale studies are needed before expanded criteria can be confidently adopted as standard selection protocol.

## 5 THE ROLE OF DOWNSTAGING

Another area of active debate is whether patients with HCC tumors exceeding accepted LT criteria at the time of diagnosis should be “downstaged” using locoregional therapies such as transarterial chemoembolization (TACE) or radiofrequency ablation (RFA) with the goal of decreasing tumor size and selecting patients where liver transplant would provide the most benefit.

Yao et al^43^ reported one of the largest series of patients with HCC undergoing downstaging to within Milan criteria/UNOS T2 criteria before LT in 2015. Patients were eligible for downstaging only if they met one of the following a priori established criteria and had absence of vascular invasion based on cross‐sectional imaging: (a) single lesion ≤8 cm; (b) two or three lesions each ≤5 cm with the sum of the maximal tumor diameters ≤8 cm; (c) four or five lesions each ≤3 cm with the sum of the maximal tumor diameters ≤8 cm. Downstaging was successful in 77 patients (65%) and 54% ultimately underwent LT. The median time from first downstaging treatment to LT was 9.8 months, which was significantly longer than the median waiting time of 8.0 months in patients within the Milan criteria. The dropout rate was 24% at 1 year and 34% at 2 years.

**Figure 5:**
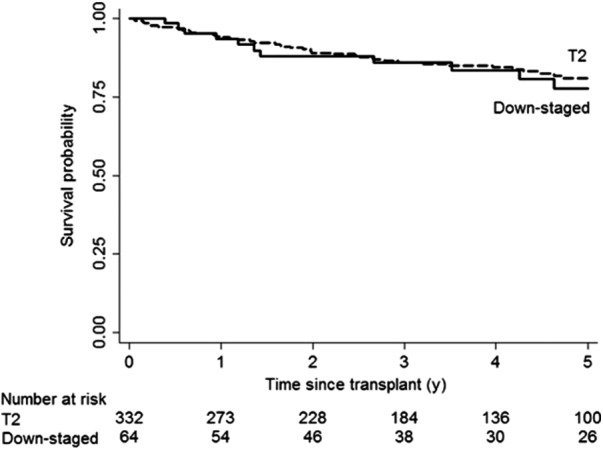
Kaplan‐Meier's post‐transplant survival probabilities of downstaging group vs T2 group from Yao et al 2015. Reprinted from Yao et al,^43^ with permission from John Wiley and Sons

Intention‐to‐treat survival outcomes at 1‐ and 5‐years, respectively, were 86% and 56% in the downstaging group vs 85% and 63% in the T2 group. The 5‐year overall survival was 78% when only those patients who were successfully downstaged were included (Figure 5). The respective 1‐ and 5‐year recurrence‐free probabilities were 95% and 91% in the downstaged group compared to 96% and 88% in the T2 group. None of these differences were statistically significant. Complete tumor necrosis with no residual tumor was observed in explant examinations of 41% of patients in the downstaging group.

In contrast, Chapman et al^44^ reported their experience with downstaging without a priori limitations. In their series, 37% of eligible patients were successfully downstaged, with 30% ultimately undergoing LT (remaining listed patients were mostly withdrawn due to death while wait‐listed or disease progression). One‐year overall survival rates between the downstaged group and within the Milan criteria group were similar (89% vs 93%); however, by the 3‐year mark these survivals began to diverge (73% vs 83%), with 5‐year overall survival of 66% vs 74%, respectively. The 5‐year recurrence rate was 11% for the downstaged group and patients falling within the Milan criteria.

A systematic review and pooled analysis of downstaging found the success rate of downstaging to be >40%.^45^ Most studies with post‐LT survival data reported 1‐year overall survival rates >90% (ranging from 87%‐100%). However, there was substantial variability in reported long‐term survival outcomes, with some studies reporting 4‐ or 5‐year survival rates >90% while others reported rates around 70%. Recurrence rates were significantly higher than in the Milan criteria group at 16%. Limitations of most of these downstaging studies included small cohort sizes, heterogeneous patient populations, and variability in downstaging protocols used. The better outcomes in the Yao et al study were likely due to stricter patient selection secondary to an a priori inclusion protocol and a mandatory observation period of 6 months prior to LT. The utility of downstaging for LT in HCC patients undoubtedly lies in the utilization of a priori limitation, selecting patients that would have more favorable outcomes following LT based on disease biology.

## 6 FUTURE DIRECTIONS

While LT selection criteria modulated on HCC tumor characteristics have been described, a major obstacle remains in creating equitable LT policies: the ability to confidently determine the risk of pre‐transplantation dropout and the true post‐transplantation benefit. Patients who have disease progression while on the waitlist are clearly at higher risk, but a subset of those patients may have tumors with more aggressive biology and, thus, if given excessive priority, may have suboptimal long‐term outcomes due to recurrence.

**Figure 6:**
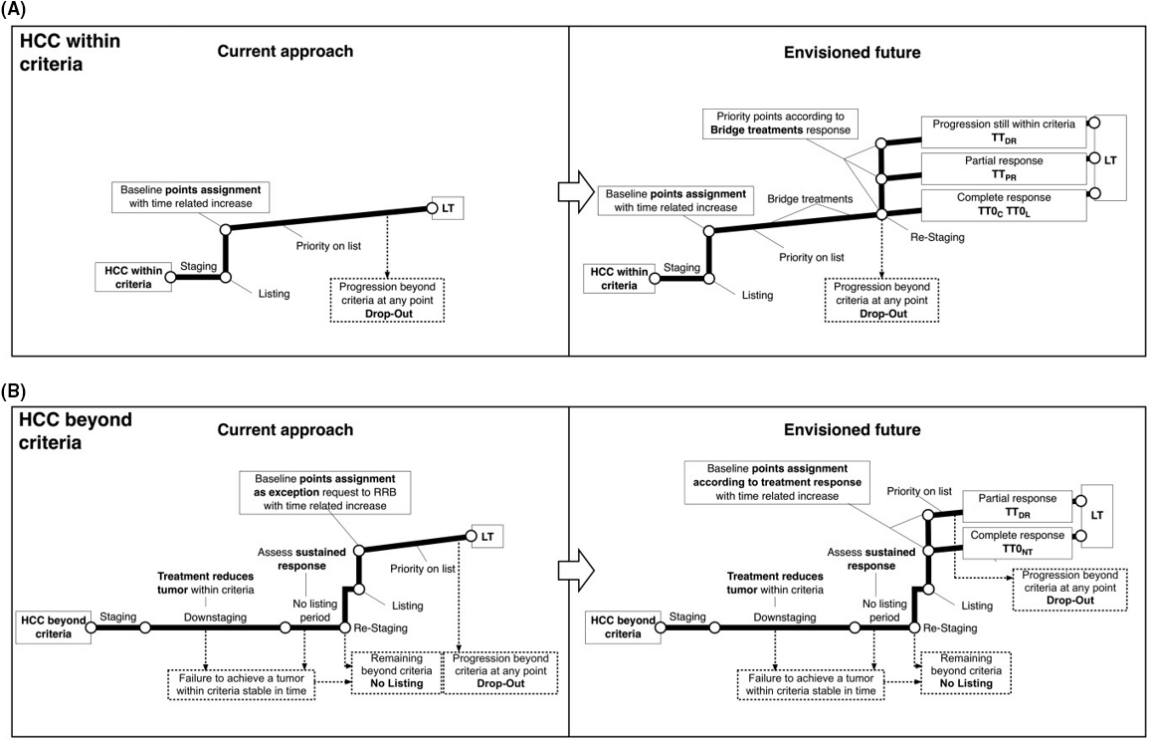
Mazzaferro et al proposed paradigm shift in management of LT in patients with HCC who are (A) within and (B) beyond criteria. Reprinted from Mazzaferro et al,^46^ with permission from John Wiley and Sons

Mazzaferro^46^ published an article in 2016 outlining an envisioned future for a multistep process from HCC diagnosis to LT in patients who are both within and beyond criteria. The proposed system utilizes tumor response to bridging or downstaging treatments as the main drivers for patient selection and allocation priority (Figure 6). He proposes that all patients with cirrhosis who have treatable HCC by non‐transplantation options should be treated upfront, regardless of whether LT is a future therapeutic option. A minimal observation period after the conclusion of a given treatment should be mandatory, as this will allow the tumor to declare its underlying tumor biology and therefore add an additional factor into the selection process. All possible information on tumor biology (AFP lab trends, tissue biopsies) should be obtained and discussed in a multi‐disciplinary setting. Finally, the minimum accepted expected survival for patients undergoing LT under these conditions should be set at 60% at 5‐years. As discussed previously in this review article, the 5‐year >60% overall survival threshold has been determined to acceptably balance LT utility and harms to non‐HCC patients on the waitlist.

## 7 CONCLUSION

Liver transplantation plays an important role in managing patients with HCC, providing the best opportunity for achieving long‐term recurrence‐free survival. Although the Milan criteria continue to be the most widely utilized transplant criteria worldwide, there is ongoing debate regarding whether the Milan criteria are too restrictive and if other expanded criteria might offer the optimal patient selection. There is also an evolving paradigm shift from “math‐based” to “biology‐based” selection criteria. Although some patients with smaller (UNOS T1 lesions) or larger (expanded criteria with or without downstaging) HCC lesions might benefit from LT, the benefits to these patients outside the commonly accepted selection criteria need to be balanced against the harms to other patients waiting for liver transplants given the limited availability of donors.

## DISCLOSURE

Funding: No sources of funding were received.

Conflict of Interest: Authors declare no conflict of interest for this article.

Author Contribution: MRJ and ACY contributed equally to the preparation of this manuscript.
